# Value of Routine Sonographic Screening of Internal Jugular Vein to Detect Catheter Related Thrombosis in Intensive Care Unit

**DOI:** 10.5005/jp-journals-10071-23207

**Published:** 2019-07

**Authors:** Manohar NM Bhat, Ramesh Venkatraman, Nagarajan Ramakrishnan, Babu K Abraham, Senthilkumar Rajagopalan

**Affiliations:** 1-5 Department of Critical Care Medicine, Apollo Hospitals, Chennai, India

**Keywords:** Central venous cannulation, CLABSI, Internal jugular vein (IJV), Sonography, Thrombosis

## Abstract

**Background and Aims:**

Internal jugular vein (IJV) cannulation is a common procedure in the ICU with thrombosis being an uncommon, albeit serious complication. Thrombosis is one of the important complications of IJV cannulation. This study aims to evaluate the use of ultrasound screening by intensivists to assess the incidence of catheter-related IJV thrombosis in ICU.

**Materials and Methods:**

Fifty consecutive IJV cannulations meeting the inclusion criteria were analyzed in the ICU. Duplex scanning and color doppler sonography were performed by the intensivist on day 3, 6, 9, 12 and 15 after cannulation. The thrombus, when detected, was confirmed independently by a radiologist. The patient demographics, the type of catheter, laterality and the mean duration of catheterization were recorded. Risk factors like presence of circulatory shock, thrombocytosis, DIC, liver disease, and absence of chemoprophylaxis for DVT were documented.

**Results:**

A total of 39 patients and 50 cannulations were studied. The mean age of patients was 56.5±16.2 years and mean duration of catheterization was 6.6±2.1 days. We found a 38% (19/50) incidence of thrombosis in our study. There was 100% correlation in detection of thrombosis by the intensivist and the radiologist. The thrombus was detected at 6.9±2.1 days after cannulation. All the patients who developed thrombosis had one or more risk factors. The most common risk factor was circulatory shock (40%). Central line associated blood stream infection (CLABSI) was seen only in the patients in whom IJV thrombus was detected (5/19).

**Conclusion:**

Catheter-related IJV thrombosis is a frequent complication in ICU patients and is associated with the increased risk of CLABSI. Ultrasound screening is simple, feasible and accurate in diagnosing IJV thrombosis.

**How to cite this article:**

Bhat MNM, Venkatraman R, Ramakrishnan N, Abraham BK, Rajagopalan S. Value of Routine Sonographic Screening of Internal Jugular Vein to Detect Catheter Related Thrombosis in Intensive Care Unit. Indian J Crit Care Med 2019;23(7):326–328.

## INTRODUCTION

Central venous cannulation is indispensable in the management of patients in the intensive care unit (ICU). Catheter-related thrombosis (CRT) has been shown to be a common complication in hemato-oncological and solid organ malignancy patients.^1^ However, there is paucity of data related to CRT and its impact on central line associated blood stream infection (CLABSI) in the general ICU population. Although the reported incidence of IJV catheter-related thrombosis in ICU varies from 33 to 67%, ^2–5^ they are rarely symptomatic. ^3,4^ In addition, it is plausible that CRT increases the risk of CLABSI.^4^

Ultrasound is commonly used clinical technique to diagnose CRT. While critical care sonography by intensivists has been established as a valuable tool for patient management,^6^ its application to detect IJV catheter-related thrombosis is yet to be studied.

The aim of this study was to determine the incidence of thrombosis associated with IJV catheters in ICU patients using routine ultrasound screening by Intensivists. As secondary outcomes, the associated risk factors and the relationship between IJV catheter related thrombosis and CLABSI were explored.

## MATERIALS AND METHODS

It was a prospective observational study conducted in an adult tertiary ICU setup. Thirty-nine patients who required IJV cannulation based on clinical indications were studied. All central venous catheters (CVC) were inserted using ultrasound guidance, and chest X-ray was done to check and adjust CVC position accordingly. All the patients received same type of catheter (ARROW®, Teleflex). Heparin coated catheters were not used. The history of malignancy and history of thrombotic disorders were the exclusion criteria. Patients who could not be followed up for 15 days following IJV cannulations were excluded from the analysis. A total of 50 cannulations were analyzed.

Strict institutional infection control protocol was practiced for placing and maintaining all the IJV catheters. IJV cannulation with both triple lumen catheters and dialysis catheters were studied using Duplex scanning and color Doppler sonography (M-Turbo, SonoSite Inc., Bothell, WA) with linear array ultrasound transducer. Sonography was done by a single intensivist on day 3, 6, 9, 12 and 15 following IJV cannulation for all patients. The thrombus, when detected, was verified by a radiologist who was blinded to the initial ultrasonography results and independently performed a sonography within 24 hours of the first evaluation. The confirmation of the presence of thrombus by the radiologist was considered as a definitive diagnosis for catheter-related thrombosis.

The patient demographics, the type of catheter, laterality of insertion and the mean duration of cannulation were noted. Presence of circulatory shock, liver disease, thrombocytosis, disseminated intravascular coagulation and absence of chemoprophylaxis for Deep Vein Thrombosis during the period of catheterization were evaluated as possible risk factors. The detection of CLABSI was also documented. Centre for Disease Control and Prevention (CDC) criteria was used for diagnosis of CLABSI ^7^. As the study was conducted as an internal audit, exemption was obtained from ethical committee.

## RESULTS

A total of 39 patients and 50 cannulations were studied (Table 1). The mean age of patients was 56.5±16.2 years and mean duration of catheterization was 6.6±2.1 days. The mean duration of catheterization in patients with and without thrombosis was 6.05 ± 1.74 and 7.03± 2.37, respectively with no significant difference (Table 2). We found 38% (19/50) incidence of thrombosis in our study. Thrombus was detected in 35% (7/20) of the left-sided cannulations and 40% (12/30) of the right-sided cannulations. Thrombosis occurred in 12 out of 34 (35%) dialysis catheter cannulations and 7 out of 16 (44%) triple lumen catheter cannulations. There was 100% correlation in detection of thrombosis between intensivist and radiologist. Thrombus was detected at 6.9±2.1 days after cannulation. While 32% of the cannulations were not associated with any of the risk factors, all the cannulations that developed thrombus had one or more risk factors during the period of catheterization. Most common risk factor found was circulatory shock (40%) followed by the absence of chemoprophylaxis for DVT (35%). Central line associated blood stream infection was associated only with the catheters in which IJV thrombosis was detected (5/19).

Pathogens responsible for CLABSI were *Klebsiella* species (n=2), *Acinetobacter* species (n=1), *Pseudomonas* species (n=1), and *Enterobacter* species (n=1).

## DISCUSSION

Central venous catheterization through IJV is a common procedure in the ICU. CRT is one of the many complications of central venous catheterization. The incidence of thrombosis has been researched extensively in patients with long-term catheters used primarily in hematological and other malignancies having the incidence variation from 1.5 to 34.1% in the former^8^ and 27% to 66% in the latter.^9^ In contrast, data regarding incidence of CRT in IJV in the intensive care setting are limited. Based on the few studies that are available, the incidence of IJV CRT is purported to be 33–67%.^2–4^

**Fig. 1:**
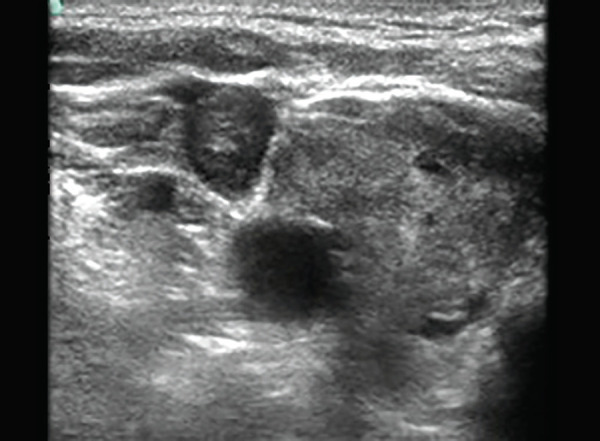
Internal jugular vein thrombosis

Contrast venography has been the gold standard for the diagnosis of CRT.^9^ But it has several disadvantages. First, it cannot be performed at patient's bedside; second, it exposes the patient to the ionizing radiations; and third is related to side effects of the contrast. Duplex scanning and color doppler sonography are a noninvasive, safe and convenient means of diagnosing CRT with good sensitivity and specificity.^8,10^ The criteria of non-compressibility of the vein (compression ultrasound) and direct visualization of the thrombotic material in the venous lumen can be used to establish the presence of thrombosis (Fig. 1). Color Doppler provides information on the blood flow. Critical care ultrasonography is a well-established bedside procedure performed by the intensivist which is safe, reliable and can be used for various indications.^11^ To our knowledge, this is the first study to evaluate the feasibility of ultrasound screening by intensivists to detect IJV catheter-related thrombosis. There was 100% correlation in the detection of thrombosis between intensivists and radiologists in our study.

The incidence of IJV CRT in our study was 38% which was similar to the incidence reported in the previous studies. There was no significant difference between the incidence of CRT among triple lumen central venous catheters and dialysis catheters. There was no difference in CRT incidence based on the laterality of insertion, which is in concordance with another study.^4^ A previous study by Timsit et al. was designed to detect thrombosis at the time of catheter removal and the mean duration of catheterization in that study was 9±5 days.^4^ A study by Rao et al. followed up for 6 days following catheterization.^3^ We designed our study based on the finding of Timsit et al.^4^ and decided to follow-up for 15 days post cannulation.

We found that CLABSI was detected only in patients who had CRT. A bidirectional relationship has been documented between CVC related infection and thrombosis ^8^by facilitating chemotherapy, supportive therapy and blood sampling. Complications of insertion of CVCs include mechanical (arterial puncture, pneumothorax.^7^ The major contributing factor for both of them is the formation of a fibrin sheath around the catheter. Previous studies performed in varied settings have also demonstrated a close relation between catheter-related central vein thrombosis and CLABSI, ^4,12,13^ increasing the risk of catheter-related sepsis by 2.6-fold. ^4^

**Table 1:** Patient demographics

Number of patients Number of cannulations Mean age Mean ICU length of stay (LOS) Mean duration of catheterization Diagnosis Sepsis Pneumonia Febrile illness (Dengue, Scrub typhus, H1N1 etc) DCLD, cholecystitis, UGI bleed Congestive heart failure Polytrauma ICH/ischemic stroke Acute pancreatitis Snake bite Bowel gangrene	39 50 56.5±16.2 yrs 16.9±17.9 days 6.6±2.1 days 28.2% 23.1% 10.3% 10.3% 5.1% 5.1% 7.7% 5.1% 2.6% 2.6%

**Table 2:** Comparison of patients with and without catheter related thrombosis

	*CRT*	*No CRT*
Number of patients	18	21
Age	51.6±11.6	60.8±18.6
Male	10	14
Female	8	7
Number of cannulations	19	31
Duration	6.05±1.74	7.03±2.37
Right sided	12	18
Left sided	7	13
Triple lumen central lines	7	9
Dialysis lines	12	22
Risk factors	1.88±0.83	1.04±1.2

Age, duration and risk factors are in mean± SD, rest in numbers

There is no standard protocol for the management of CRT in ICU patients. The treatment strategies include thrombolytic therapy, initiation of systemic anticoagulation, and removal of the catheter. Based on the guidelines for the treatment of CRT in oncological patients, prolonged anticoagulation with LMWH with changeover to vitamin K antagonists may be considered.^14,15^ There is no recommendation for thrombolytic therapy and catheter removal to treat CRT in ICU patients. However, since CRT is strongly associated with CLABSI, it would be prudent to actively screen for catheter-related thrombosis in patients with IJV catheters and remove the catheter whenever the thrombus is detected. More studies are required in the ICU setting to formulate therapeutic strategies for the prevention and management of CRT.

Our study is limited in that it is single centered and only IJV catheters were evaluated. The incidence and risk of CRT may differ at other sites. These rates may not reflect in other centers as well. We requested an independent radiologist confirmation of IJV thrombus by ultrasonography only in patients who had positive screening upon examination. Hence, we could not decipher false negative rates of the bedside ultrasonography evaluation of IJV thrombus. Also, DVT prophylaxis practice was not protocolized; 65% of our patients received chemoprophylaxis for prevention of DVT. This might have decreased the incidence of thrombosis in our study. The strengths of our study include rigorous protocolized screening of all IJV catheters, objective diagnosis of IJV thrombosis, independent confirmation of the IJV thrombus by a radiologist blinded to the initial ultrasonography results, and correlation with the common risk factors.

## CONCLUSIONS

Incidence of thrombosis associated with IJV catheters in our study was similar to the incidence in the previous studies. The incidence rates between triple lumen and dialysis catheters are similar in our study. CVC related thrombosis seems to be associated with a higher risk of CLABSI. We found that circulatory shock is the commonest risk factor associated with CRT routine. Ultrasound screening of IJV catheters by intensivists to evaluate CRT is recommended.
